# Is it essential for the combination of GOLD C and D groups in COPD patients: A multicenter retrospective study?

**DOI:** 10.1097/MD.0000000000050492

**Published:** 2026-08-28

**Authors:** Tao Li, Ping Zhang, Qing Song, Cong Liu, Ling Lin, Yuqin Zeng, Ping Chen

**Affiliations:** a Department of Pulmonary and Critical Care Medicine, The Second Xiangya Hospital, Central South University, Changsha, Hunan, China; b Research Unit of Respiratory Disease, Central South University, Changsha, Hunan, China; c Clinical Medical Research Center for Pulmonary and Critical Care Medicine, Changsha, Hunan, China; d Diagnosis and Treatment Center of Respiratory Disease, Central South University, Changsha, Hunan, China.

**Keywords:** COPD, exacerbation, GOLD, mortality, treatment

## Abstract

The global initiative for chronic obstructive lung disease (GOLD) 2023 combined the C and D groups into the E group. This study aimed to explore the necessity of combining the GOLD C and D groups based on prognosis and inhaled treatment response in patients with chronic obstructive pulmonary disease (COPD). This study retrospectively analyzed data from the Real-world Diapnosis and Treatment of COPD cohort study from January 2017 to June 2022. The baseline demographic and clinical data were organized. According to the GOLD 2017, patients were classified into 4 groups (A, B, C, and D). At follow-up, the number of moderate-to-severe exacerbations in 1 year and all-cause mortality in 3 years were recorded. A total of 4970 COPD patients were included in this study, with a mean age of 62.4 ± 8.4 years; 86.9% were male, and 77.6% were smokers. The GOLD C group included 156 (3.1%) patients. Logistic regression with stratified adjustment showed that patients in the GOLD A and B groups had the lowest risk of future exacerbation (A group: odds ratio [OR] = 0.369, 95% confidence interval [CI] = 0.249–0.546; B group: OR = 0.447, 95% CI = 0.339–0.671), frequent exacerbation (A group: OR = 0.291, 95% CI = 0.169–0.503; B group: OR = 0.458, 95% CI = 0.296–0.709), and hospitalization (A group: OR = 0.349, 95% CI = 0.219–0.556; B group: OR = 0.394, 95% CI = 0.267–0.582), whereas patients in the GOLD D group had similar risk values to patients in the GOLD C group. Stratified logistic regression also showed that different inhaled treatments had no impact on the risk of future exacerbation, frequent exacerbation, and hospitalization in the GOLD C group, while in GOLD D group, long-acting β-2-agonist/long-acting muscarinic antagonist (LABA/LAMA) or LABA/LAMA/inhaled corticosteroids could decrease the risk of future any exacerbation and frequent exacerbation but the risk of hospitalization was similar among different inhaled treatment groups. The GOLD C group, as the smallest group, showed a similar prognosis to the GOLD D group. The effect of different inhaled treatments was almost the same in the GOLD C group, while LABA/LAMA and LABA/LAMA/inhaled corticosteroids were superior for patients in the GOLD D group.

## 1. Introduction

Chronic obstructive pulmonary disease (COPD) was the 3rd leading cause of death worldwide in 2019.^[1]^ In China, the prevalence of COPD patients aged ≥40 years is 13.7%,^[2]^ which is a huge health and economic burden. The global initiative for chronic obstructive lung disease (GOLD) was established in 1998, and its 1st report was published in 2001. It is currently an important reference for physicians in diagnosing and treating patients with COPD.

The GOLD 2017 suggested classifying COPD patients into A, B, C, and D groups based on symptom evaluation and exacerbation history. The COPD assessment test (CAT) and modified Medical Research Council (mMRC) dyspnea scale (hereinafter referred to as mMRC) were suggested for evaluating COPD symptoms according to the GOLD guidelines. The mMRC was used to assess dyspnea in patients with COPD, as several studies have demonstrated that it is an independent predictor of future exacerbations and mortality.^[3–8]^ The CAT included 8 symptom domains, such as cough, phlegm, and breathlessness, and thus could provide a more comprehensive symptom score for COPD patients. Several studies have shown that the CAT at baseline was associated with the risk of exacerbation and mortality, and that changes in the CAT were superior to the baseline CAT for predicting prognosis.^[7,9–13]^ Additionally, exacerbation history was found to be the best predictor for future exacerbation in COPD.^[14,15]^ However, the GOLD 2023 proposed replacing the C and D groups with the E group, focusing less on symptoms in high-exacerbation-risk patients, and recommended long-acting β-2-agonist (LABA)/long-acting muscarinic antagonist (LAMA) as the initial treatment for patients in the E group. Duckworth et al^[16]^ observed that patients in the GOLD C group had a lower exacerbation rate and CAT score than those in the GOLD D group after 1 year follow-up, with the suggestion that the proposal of the E group should be reconsidered. Given that the GOLD is an important reference for clinical doctors treating COPD patients, it is valuable to explore the rationale for combining the C and D groups. Other studies have shown that compared to LABA/inhaled corticosteroids (ICS) or LAMA, LABA/LAMA can reduce the exacerbation and hospitalization risk in COPD patients; this result was found overall in patients with COPD,^[17–20]^ and it is not clear whether this positive trend of LABA/LAMA is the same in the GOLD C or D groups.

Therefore, this study investigated whether the risks of future exacerbation and all-cause mortality were the same between the GOLD C and D groups and simultaneously analyzed whether there were differences in future exacerbation risk among different inhaled treatments in the GOLD C and D groups.

## 2. Methods

### 2.1. Study participants and design

The participants in this study were from the RealDTC (ChiCTR-POC-17010431) study.^[21]^ This study analyzed data from January 2017 to June 2022, including patients diagnosed with COPD (according to the GOLD 2017 report, patients with forced expiratory volume in 1 second [FEV_1_]/forced vital capacity <70% after bronchodilator inhalation were diagnosed with COPD), aged ≥40 years, and excluding patients with mental disorders. This study was approved by the Ethics Committee of the Second Xiangya Hospital (2016076). Written informed consent was obtained from all patients.

### 2.2. Data collection

The baseline data, including age, gender, educational level, height, weight, CAT, mMRC, FEV_1_% predicted, FEV_1_/forced vital capacity, smoking pack/year, occupational exposure, biofuel exposure, exacerbation history, inhaled treatment and comorbidities (including asthma, bronchiectasis, pulmonary tuberculosis, cardiovascular diseases, and diabetes), were collected. Cardiovascular diseases included cardiomyopathy, coronary heart disease, hypertension, heart valve disease, arrhythmia, and related conditions. At follow-up, the number of moderate-to-severe exacerbations in 1 year and all-cause mortality in 3 years were recorded. All patients received the same inhaled treatment during follow-up, and all patients ultimately included in this study had clear records of the number of moderate-to-severe exacerbations in 1 year and survival status at 3 years.

Smokers were defined as those who had smoked >10 packs/year in their lifetime; current smokers were defined as those who had not quit smoking or had quit smoking for <6 months, while former smokers were defined as those who had quit smoking for ≥6 months; otherwise, they were defined as never smoked.^[22]^ Occupational exposure was defined as exposure to dust, metals, chemical substances, or other environmental agents for at least 1 year and at least 8 hours a day.^[23]^ Biofuel exposure was defined as the use of biomass fuels (wood, grass, charcoal, and crop residues) for cooking or heating for at least 1 year and for at least 2 hours/d.^[24]^

### 2.3. Variable definition

In this study, exacerbation was defined as the acute deterioration of respiratory symptoms that required additional treatment, including corticosteroid and/or antibiotic treatment (moderate exacerbation) and hospitalization (severe exacerbation).^[25]^ Frequent exacerbations were defined as at least 2 exacerbations/yr.^[26]^ Hospitalization was defined as a COPD patient’s admission due to exacerbation. All-cause mortality was defined as the death of a COPD patient due to any disease or accident during the years of follow-up.

According to the GOLD 2017, patients were classified into 4 groups:

A group, with 0 to 1 exacerbation/yr, no hospitalization, CAT <10, and mMRC of 0 to 1;
B group, with 0 to 1 exacerbation/yr, no hospitalization, CAT ≥10 or mMRC ≥2;
C group, with ≥2 exacerbations or ≥1 hospitalization per year, CAT <10 and mMRC of 0 to 1;
D group, with ≥2 exacerbations or ≥1 hospitalization per year, CAT ≥10 or mMRC ≥2.

The GOLD C and D groups were then classified into LAMA, LABA/ICS, LABA/LAMA, and LABA/LAMA/ICS groups according to inhaled treatment.

### 2.4. Data wwanalysis

SPSS 26.0 (IBM) was used for statistical analysis in this study. Continuous variables are presented as mean ± standard deviation or median and interquartile range. The chi-square test was used to analyze whether there were differences in the rates of future exacerbation and all-cause mortality between the GOLD C and A, B, or D groups. Logistic regression and COX regression were used to analyze the risks of future exacerbation and all-cause mortality, respectively, between the GOLD C and A, B, or D groups, adjusting for confounding factors via stratification. The chi-square test was used to analyze whether there were differences in the rate of future exacerbations among inhaled treatments in the GOLD C and D groups. Logistic regression was used to analyze exacerbation risk across different inhaled treatments in the GOLD C and D groups, adjusting for confounding factors through stratification.

## 3. Results

### 3.1. Demographic and clinical characteristics of COPD patients

**Figure 1.**
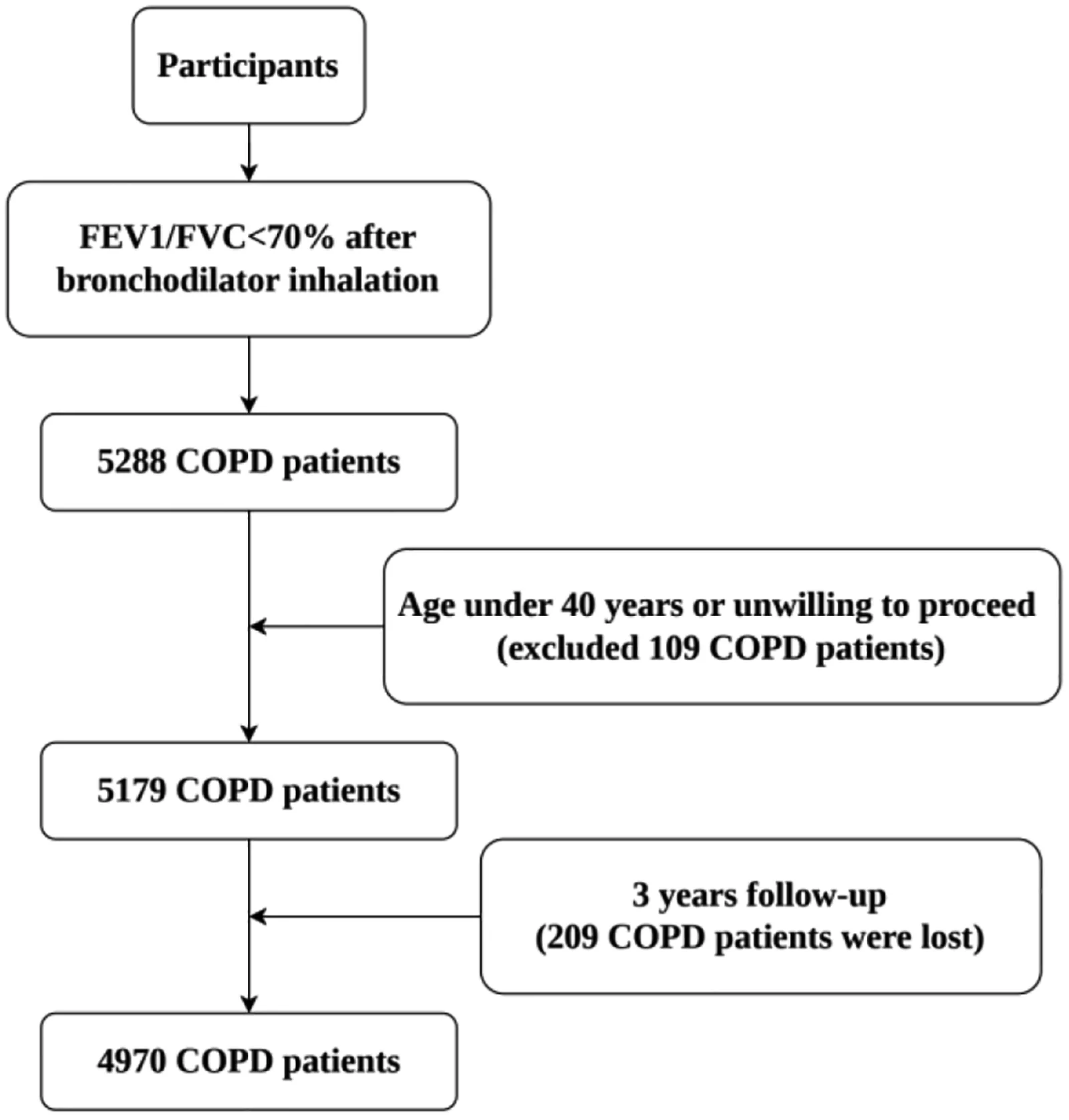
Flowchart. COPD = chronic obstructive pulmonary disease, FEV_1_ = forced expiratory volume in 1 second, FVC = forced vital capacity.

According to the COPD diagnosis, 5288 patients were included at baseline. Due to age <40, unwillingness to participate, and loss to follow-up, 318 COPD patients were excluded, and 4970 COPD patients were ultimately enrolled; all patients had complete data on future exacerbations and all-cause mortality (Fig. 1).

The average age of participants was 62.4 ± 8.4, with 86.9% male and 77.6% smokers. The mean body mass index, CAT, and FEV1% predicted were 22.7 ± 3.5, 14.9 ± 6.5, and 54.2 ± 20.2, respectively. Almost 49.9% of COPD patients had an exacerbation history (at least 1 moderate-to-severe exacerbation in the past 1 year). The distribution of the GOLD groups was 543 (10.9%) in the A group, 2353 (47.4%) in the B group, 156 (3.1%) in the C group, and 1918 (38.6%) in the D group (Table 1).

**Table 1:** Characteristics of COPD patients at baseline.

Characteristics	Total (N = 4970)
Age (mean ± SD)	62.4 ± 8.4
Gender	
Male	4317 (86.9)
Female	653 (13.1)
Smoking pack-year (median, IQR)	35 (12.1, 50.0)
BMI (mean ± SD)	22.7 ± 3.5
CAT (mean ± SD)	14.9 ± 6.5
mMRC (median, IQR)	2.0 (1.0, 3.0)
FEV_1_% predicted (mean ± SD)	54.2 ± 20.2
FEV_1_/FVC (mean ± SD)	48.0 ± 12.0
Educational level	
Junior school and below	3900 (78.5)
High school and above	1070 (21.5)
Smoking status	
Nonsmoker	1111 (22.4)
Former smoker	1642 (33.0)
Current smoker	2217 (44.6)
Occupational exposure	
No	3076 (61.9)
Yes	1894 (38.1)
Biofuel exposure	
No	3279 (66.0)
Yes	1691 (34.0)
The exacerbation history	
No	2489 (50.1)
Yes	2481 (49.9)
GOLD 2017	
A	543 (10.9)
B	2353 (47.4)
C	156 (3.1)
D	1918 (38.6)
Inhaled treatment	
LAMA	1436 (28.9)
LABA/ICS	673 (13.5)
LABA/LAMA	523 (10.5)
LABA/LAMA/ICS	2338 (47.1)
Comorbidity	
Asthma	1082 (21.8)
Bronchiectasis	197 (4.0)
Pulmonary tuberculosis	86 (1.7)
Cardiovascular diseases	188 (3.8)
Diabetes	33 (0.7)

Cardiovascular diseases include cardiomyopathy, coronary heart disease, hypertension, heart valve disease, arrhythmia and others.

BMI = body mass index, CAT = COPD assessment test, COPD = chronic obstructive pulmonary diseases, FEV1 = forced expiratory volume in 1 second, FVC = forced vital capacity, GOLD = global initiative for chronic obstructive lung disease, ICS = inhaled corticosteroids, IQR = interquartile range, LABA = long-acting β-2-agonist, LAMA = long-acting muscarinic antagonist, mMRC = modified medical research council dyspnea scale, SD = standard deviation.

### 3.2. Comparison between GOLD C and A, B or D groups for the rates or risks of future exacerbation and all-cause mortality

**Figure 2.**
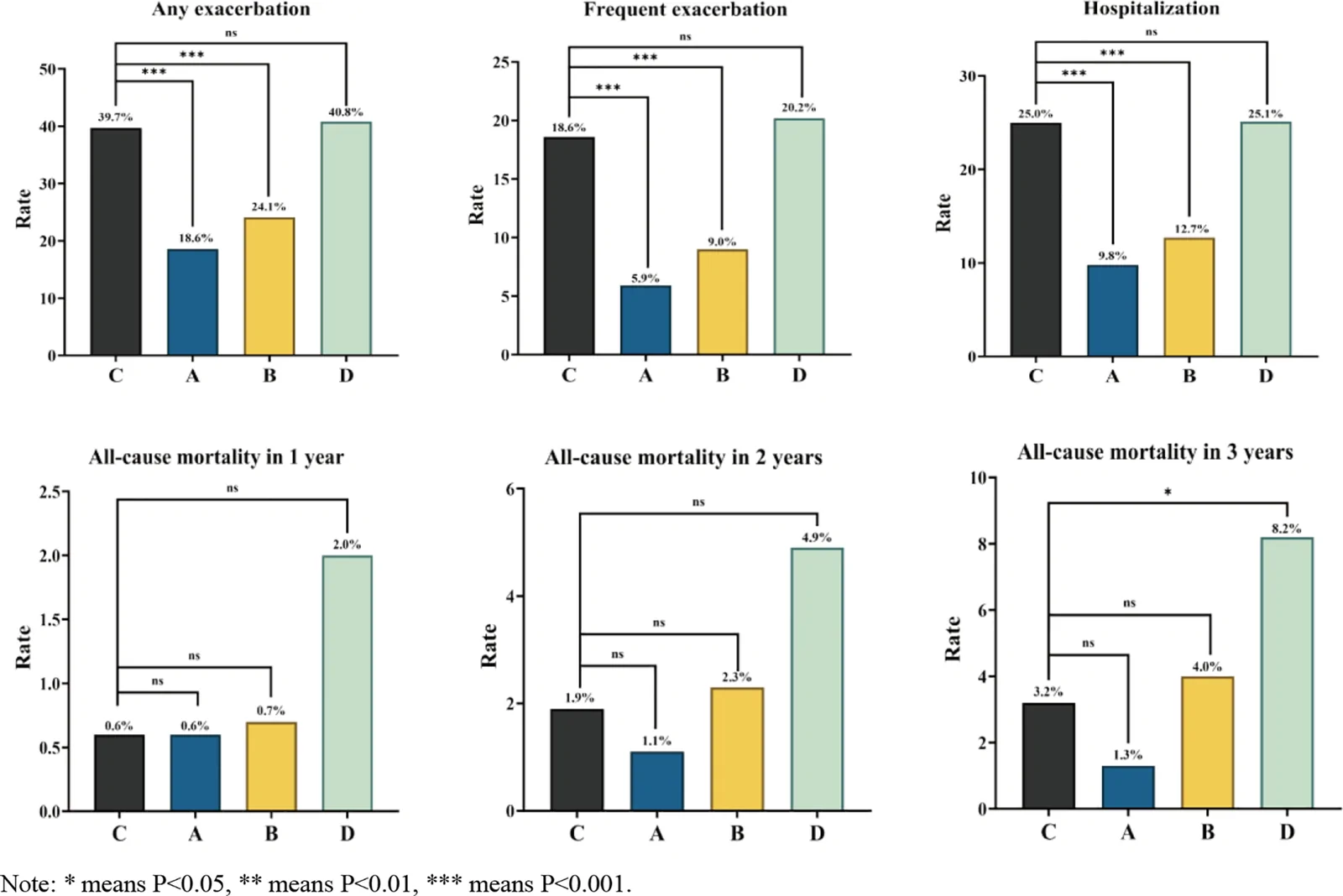
Comparison between GOLD 2017 C and A, B or D groups for the rates of future exacerbation and all-cause mortality. GOLD = global initiative for chronic obstructive lung disease.

Regarding future exacerbation, patients in the GOLD C group suffered the highest rates of any future exacerbation, frequent exacerbation, and hospitalization than those in the GOLD A and B groups, but exhibited similar rates when compared to patients in the GOLD D group (Fig. 2). Further analysis by logistic regression showed that no matter how many confounding factors were included, patients in the GOLD A and B groups had a lower risk of any future exacerbation (A group: odds ratio [OR] = 0.369, 95% confidence interval [CI] = 0.249–0.546; B group: OR = 0.447, 95% CI = 0.339–0.671), frequent exacerbation (A group: OR = 0.291, 95% CI = 0.169–0.503; B group: OR = 0.458, 95% CI = 0.296–0.709), and hospitalization (A group: OR = 0.349, 95% CI = 0.219–0.556; B group: OR = 0.394, 95% CI = 0.267–0.582), while patients in the GOLD D group showed no difference in these statistics compared to patients in the GOLD C group (Table 2).

**Table 2:** Comparison between GOLD 2017 C and A, B or D groups for future exacerbation by logistic regression analysis.

Model 1	Any exacerbation		Frequent exacerbation		Hospitalization	
	OR (95% CI)	*P* value	OR (95% CI)	*P* value	OR (95% CI)	*P* value
C group	Reference		Reference		Reference	
A group	0.346 (0.235–0.510)	<.001	0.274 (0.160–0.470)	<.001	0.324 (0.205–0.514)	<.001
B group	0.480 (0.344–0.671)	<.001	0.431 (0.281–0.661)	<.001	0.435 (0.297–0.638)	<.001
D group	1.044 (0.748–1.457)	.802	1.111 (0.731–1.688)	.623	1.007 (0.691–1.468)	.971

Model 2	Any exacerbation		Frequent exacerbation		Hospitalization	
	OR (95% CI)	*P* value	OR (95% CI)	*P* value	OR (95% CI)	*P* value
C group	Reference		Reference		Reference	
A group	0.361 (0.245–0.533)	<.001	0.284 (0.165–0.490)	<.001	0.340 (0.214–0.541)	<.001
B group	0.468 (0.334–0.657)	<.001	0.450 (0.292–0.696)	<.001	0.385 (0.261–0.567)	<.001
D group	0.973 (0.691–1.368)	.873	1.116 (0.726–1.717)	.617	0.842 (0.572–1.240)	.384

Model 3	Any exacerbation		Frequent exacerbation		Hospitalization	
	OR (95% CI)	*P* value	OR (95% CI)	*P* value	OR (95% CI)	*P* value
C group	Reference		Reference		Reference	
A group	0.369 (0.249–0.546)	<.001	0.291 (0.169–0.503)	<.001	0.349 (0.219–0.556)	<.001
B group	0.477 (0.339–0.671)	<.001	0.458 (0.296–0.709)	<.001	0.394 (0.267–0.582)	<.001
D group	0.998 (0.708–1.406)	.990	1.142 (0.741–1.759)	.548	0.869 (0.589–1.283)	.480

The model 1 included GOLD groups.

The model 2 included GOLD groups, age, gender, BMI, FEV_1_% predicted, smoking status, biofuel exposure, occupational exposure, educational level and inhaled treatment.

The model 3 included GOLD groups, age, gender, BMI, FEV_1_% predicted, smoking status, biofuel exposure, occupational exposure, educational level, inhaled treatment and comorbidities.

BMI = body mass index, CI = confidence interval, FEV_1_ = forced expiratory volume in 1 second, GOLD = global initiative for chronic obstructive lung disease, OR = odds ratio.

**Figure 3.**
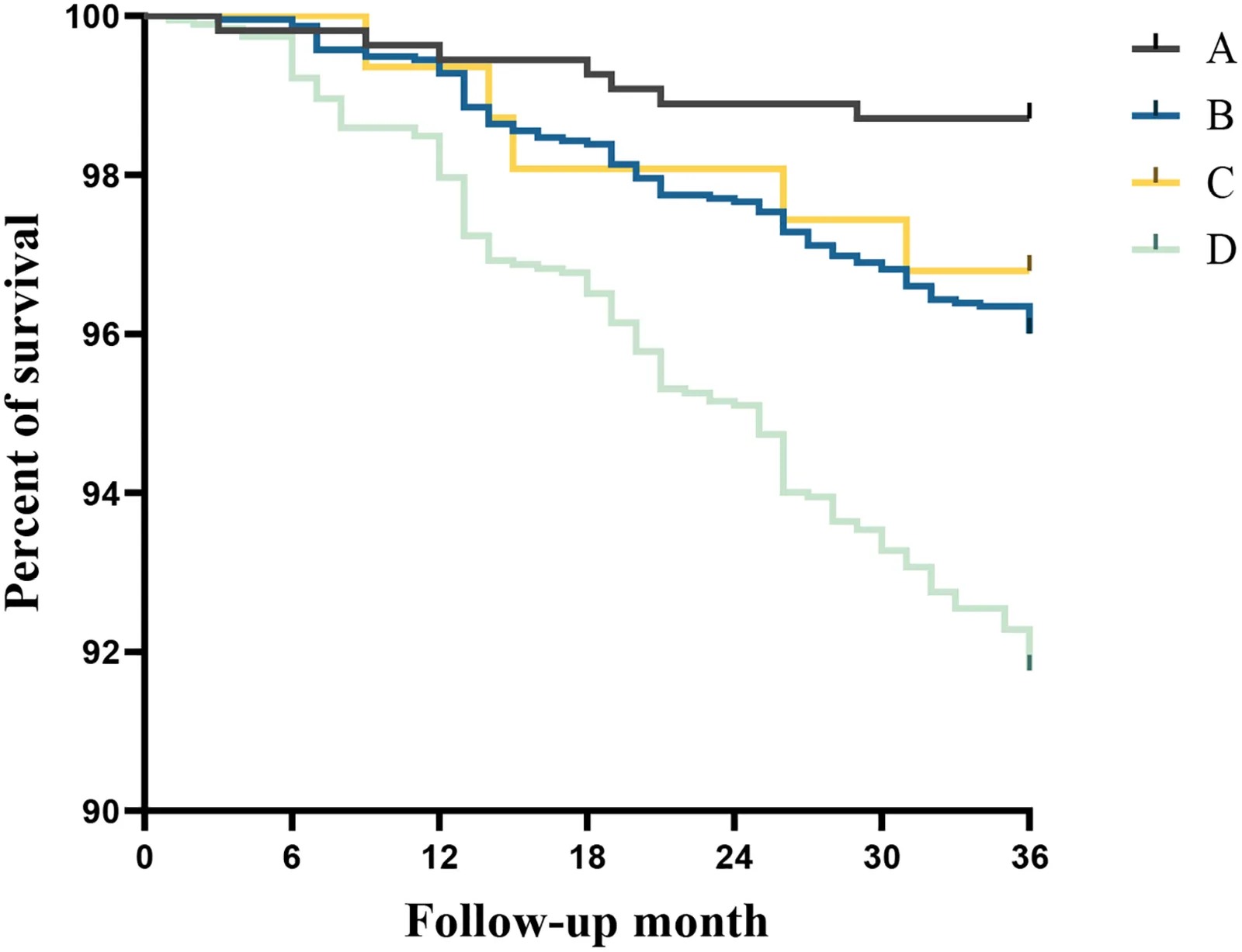
Kaplan–Meier survival curve for GOLD 2017 groups in 3-year follow-up. GOLD = global initiative for chronic obstructive lung disease.

For long-term prognosis, all-cause mortality was recorded as 1.3% in the A group, 4.0% in the B group, 3.2% in the C group, and 8.2% in the D group at 3-year follow-up. However, there was no statistical difference in all-cause mortality between the GOLD C and A, B, or D groups, except that patients in the GOLD D group had a higher rate of 3-year all-cause mortality than patients in the GOLD C group (Fig. 2). COX regression with adjustment for stratification showed similar trends of risk between the GOLD C and A, B, or D groups in all-cause mortality, but the GOLD D group (OR = 2.634, 95% CI = 1.081–6.415) had a higher risk of 3-year all-cause mortality than the GOLD C group by univariate COX regression (Table 3). Moreover, the GOLD C group showed greater instability in the Kaplan–Meier survival curve than the other GOLD groups (Fig. 3).

**Table 3:** Comparison between GOLD 2017 C and A, B or D groups for all-cause mortality by COX regression analysis.

Model 1	All-cause mortality in 1 yr		All-cause mortality in 2 yr		All-cause mortality in 3 yr	
	OR (95% CI)	*P* value	OR (95% CI)	*P* value	OR (95% CI)	*P* value
C group	Reference		Reference		Reference	
A group	0.862 (0.090–8.288)	.898	0.572 (0.143–2.287)	.572	0.399 (0.127–1.257)	.399
B group	1.128 (0.150–8.476)	.907	1.216 (0.380–3.887)	.741	1.249 (0.508–3.071)	.628
D group	3.194 (0.439–23.249)	.251	2.585 (0.819–8.159)	.105	2.634 (1.081–6.415)	.033

Model 2	All-cause mortality in 1 yr		All-cause mortality in 2 yr		All-cause mortality in 3 yr	
	OR (95% CI)	*P* value	OR (95% CI)	*P* value	OR (95% CI)	*P* value
C group	Reference		Reference		Reference	
A group	0.965 (0.100–9.337)	.975	0.661 (0.165–2.653)	.559	0.464 (0.147–1.464)	.190
B group	0.928 (0.123–7.027)	.943	1.009 (0.314–3.243)	.988	0.960 (0.389–2.372)	.930
D group	2.449 (0.331–18.125)	.380	1.899 (0.594–6.067)	.279	1.707 (0.694–4.200)	.244

Model 3	All-cause mortality in 1 yr		All-cause mortality in 2 yr		All-cause mortality in 3 yr	
	OR (95% CI)	*P* value	OR (95% CI)	*P* value	OR (95% CI)	*P* value
C group	Reference		Reference		Reference	
A group	1.051 (0.107–10.330)	.966	0.864 (0.214–3.486)	.837	0.529 (0.167–1.675)	.279
B group	0.905 (0.118–6.910)	.923	1.179 (0.365–3.809)	.783	1.036 (0.417–2.575)	.939
D group	2.519 (0.336–18.859)	.368	2.308 (0.717–7.430)	.161	1.943 (0.784–4.818)	.152

The model 1 included GOLD groups.

The model 2 included GOLD groups, age, gender, BMI, FEV_1_% predicted, smoking status, biofuel exposure, occupational exposure, educational level and inhaled treatment.

The model 3 included GOLD groups, age, gender, BMI, FEV_1_% predicted, smoking status, biofuel exposure, occupational exposure, educational level, inhaled treatment and comorbidities.

BMI = body mass index, CI = confidence interval, FEV_1_ = forced expiratory volume in 1 second, GOLD = global initiative for chronic obstructive lung disease, OR = odds ratio.

### 3.3. Inhaled treatment response in the GOLD C and D groups

Inhaled treatment is the core of COPD therapy. Compared to the GOLD C group, there were more patients treated with LAMA but fewer patients treated with LABA/LAMA/ICS in the GOLD A group, while there were fewer patients treated with LAMA but more patients treated with LABA/LAMA/ICS in the GOLD B and D groups (Table 4).

**Table 4:** Distribution of inhaled treatment in different GOLD 2017 groups.

Groups	LAMA	LABA/ICS	LABA/LAMA	LABA/LAMA/ICS	*P* value
C group	49 (31.4)	38 (24.4)	16 (10.2)	53 (34.0)	
A group	230 (42.4)	100 (18.4)	77 (14.2)	136 (25.0)	.013
B group	647 (27.5)	331 (14.1)	278 (11.8)	1097 (46.6)	<.001
D group	510 (26.6)	204 (10.6)	152 (7.9)	1052 (54.9)	<.001

*P* value means that C group was compared to A, B or D groups, respectively.

GOLD = global initiative for chronic obstructive lung disease, ICS = inhaled corticosteroids, LABA = long-acting β-2-agonist, LAMA = long-acting muscarinic antagonist.

**Figure 4.**
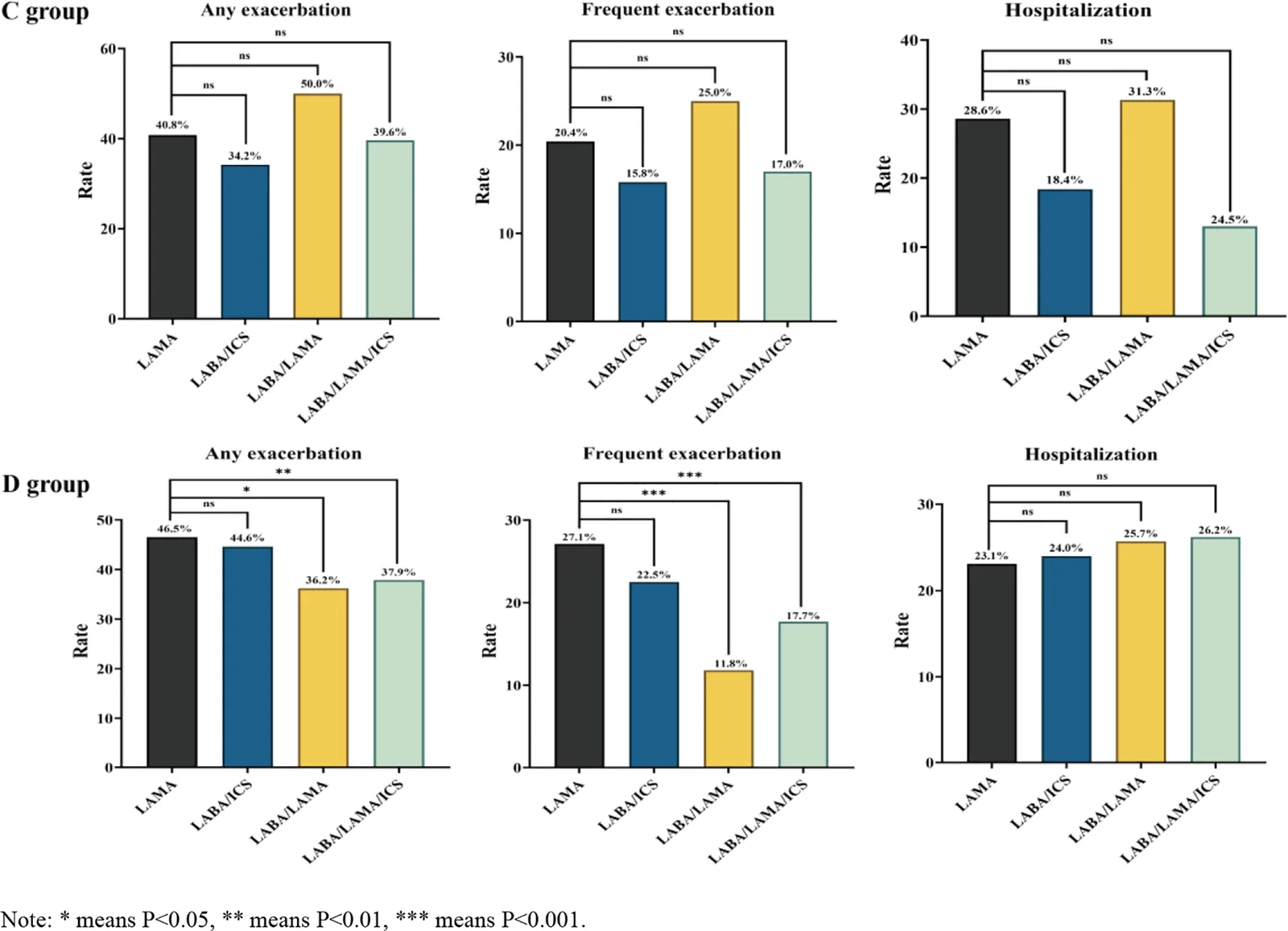
Comparison among different inhaled treatment groups in GOLD 2017 C and D groups for the rate of future exacerbation. GOLD = global initiative for chronic obstructive lung disease, ICS = inhaled corticosteroids, LABA = long-acting β-2-agonist, LAMA = long-acting muscarinic antagonist.

In the GOLD C group, the rates of any future exacerbation, frequent exacerbation, and hospitalization showed similar trends across inhaled treatment groups (Fig. 4). Further analysis using stratified logistic regression also showed that different inhaled treatments did not change the risk of future exacerbation, frequent exacerbation, or hospitalization in the GOLD C group (Table 5).

**Table 5:** Comparison among different inhaled treatment in GOLD 2017 C group for future exacerbation by logistic regression analysis.

Model 1	Any exacerbation		Frequent exacerbation		Hospitalization	
	OR (95% CI)	*P* value	OR (95% CI)	*P* value	OR (95% CI)	*P* value
LAMA	Reference		Reference		Reference	
LABA/ICS	0.754 (0.313–1.817)	.529	0.731 (0.240–2.230)	.582	0.565 (0.202–1.578)	.276
LABA/LAMA	1.450 (0.467–4.505)	.521	1.300 (0.345–4.905)	.699	1.136 (0.334–3.870)	.838
LABA/LAMA/ICS	0.952 (0.431–2.101)	.902	0.798 (0.294–2.165)	.657	0.813 (0.337–1.960)	.644

Model 2	Any exacerbation		Frequent exacerbation		Hospitalization	
	OR (95% CI)	*P* value	OR (95% CI)	*P* value	OR (95% CI)	*P* value
LAMA	Reference		Reference		Reference	
LABA/ICS	0.662 (0.258–1.700)	.391	0.669 (0.204–2.191)	.506	0.455 (0.147–1.408)	.172
LABA/LAMA	1.427 (0.406–5.008)	.579	1.208 (0.290–5.036)	.795	0.839 (0.207–3.407)	.806
LABA/LAMA/ICS	0.825 (0.335–2.033)	.676	0.716 (0.235–2.179)	.557	0.743 (0.268–2.058)	.567

Model 3	Any exacerbation		Frequent exacerbation		Hospitalization	
	OR (95% CI)	*P* value	OR (95% CI)	*P* value	OR (95% CI)	*P* value
LAMA	Reference		Reference		Reference	
LABA/ICS	0.735 (0.251–2.155)	.575	0.485 (0.119–1.987)	.315	0.897 (0.224–3.587)	.878
LABA/LAMA	1.666 (0.457–6.075)	.439	1.158 (0.271–4.945)	.843	0.349 (0.101–1.208)	.097
LABA/LAMA/ICS	0.951 (0.321–2.813)	.927	0.519 (0.135–2.000)	.341	0.597 (0.198–1.800)	.359

The model 1 included inhaled treatment.

The model 2 included inhaled treatment, age, gender, BMI, FEV_1_% predicted, smoking status, biofuel exposure, occupational exposure, and educational level.

The model 3 included inhaled treatment, age, gender, BMI, FEV_1_% predicted, smoking status, biofuel exposure, occupational exposure, educational level and comorbidities.

BMI = body mass index, CI = confidence interval, FEV_1_ = forced expiratory volume in 1 second, GOLD = global initiative for chronic obstructive lung disease, ICS = inhaled corticosteroids, LABA = long-acting β-2-agonist, LAMA = long-acting muscarinic antagonist, OR = odds ratio.

In the GOLD D group, patients treated with LABA/LAMA or LABA/LAMA/ICS had lower rates of future and frequent exacerbations compared to patients treated with LAMA, but future hospitalization rates were similar across inhaled treatment groups (Fig. 4). Logistic regression with stratified adjustment also demonstrated that LABA/LAMA or LABA/LAMA/ICS could decrease the risk of any future exacerbation (LABA/LAMA: OR = 0.623, 95% CI = 0.427–0.909; LABA/LAMA/ICS: OR = 0.665, 95% CI = 0.531–0.832) and frequent exacerbation (LABA/LAMA: OR = 0.350, 95% CI = 0.205–0.599; LABA/LAMA/ICS: OR = 0.531, 95% CI = 0.406–0.693), but the risk of hospitalization was similar among the different inhaled treatment groups (Table 6).

**Table 6:** Comparison among different inhaled treatments in GOLD 2017 D group for future exacerbation by logistic regression analysis.

Model 1	Any exacerbation		Frequent exacerbation		Hospitalization	
	OR (95% CI)	*P* value	OR (95% CI)	*P* value	OR (95% CI)	*P* value
LAMA	Reference		Reference		Reference	
LABA/ICS	0.928 (0.669–1.286)	.652	0.785 (0.536–1.150)	.214	1.050 (0.717–1.538)	.801
LABA/LAMA	0.653 (0.449–0.949)	.026	0.362 (0.213–0.615)	<.001	1.147 (0.755–1.742)	.522
LABA/LAMA/ICS	0.704 (0.568–0.872)	.001	0.579 (0.450–0.645)	<.001	1.182 (0.923–1.513)	.186

Model 2	Any exacerbation		Frequent exacerbation		Hospitalization	
	OR (95% CI)	*P* value	OR (95% CI)	*P* value	OR (95% CI)	*P* value
LAMA	Reference		Reference		Reference	
LABA/ICS	0.954 (0.687–1.325)	.778	0.825 (0.560–1.216)	.331	1.100 (0.748–1.619)	.627
LABA/LAMA	0.620 (0.425–0.904)	.013	0.343 (0.200–0.586)	<.001	1.050 (0.687–1.603)	.823
LABA/LAMA/ICS	0.666 (0.533–0.834)	<.001	0.540 (0.414–0.704)	<.001	1.161 (0.895–1.505)	.260

Model 3	Any exacerbation		Frequent exacerbation		Hospitalization	
	OR (95% CI)	*P* value	OR (95% CI)	*P* value	OR (95% CI)	*P* value
LAMA	Reference		Reference		Reference	
LABA/ICS	0.953 (0.685–1.324)	.772	0.813 (0.551–1.200)	.297	1.064 (0.715–1.583)	.760
LABA/LAMA	0.623 (0.427–0.909)	.014	0.350 (0.205–0.599)	<.001	1.032 (0.675–1.579)	.884
LABA/LAMA/ICS	0.665 (0.531–0.832)	<.001	0.531 (0.406–0.693)	<.001	1.109 (0.839–1.465)	.467

The model 1 included inhaled treatment.

The model 2 included inhaled treatment, age, gender, BMI, FEV_1_% predicted, smoking status, biofuel exposure, occupational exposure, and educational level.

The model 3 included inhaled treatment, age, gender, BMI, FEV_1_% predicted, smoking status, biofuel exposure, occupational exposure, educational level, and comorbidities.

BMI = body mass index, CI = confidence interval, FEV_1_ = forced expiratory volume in 1 second, GOLD = global initiative for chronic obstructive lung disease, ICS = inhaled corticosteroids, LABA = long-acting β-2-agonist, LAMA = long-acting muscarinic antagonist, OR = odds ratio.

## 4. Discussion

This study showed no statistically significant difference between the GOLD C and D groups in the risk of future exacerbation and all-cause mortality. Patients in the GOLD C group suffered a similar risk of future exacerbation among the different inhaled treatment groups. Still, LABA/LAMA or LABA/LAMA/ICS reduced the risk of future exacerbation in patients of the GOLD D group, which suggests that the combination of the GOLD C and D groups could be considered.

To the best of our knowledge, the GOLD C group was the smallest, ranging from 1.3% to 6.9%^[27–33]^ and in this study, the proportion of the GOLD C group was 3.1%, consistent with previous studies. The GOLD 2023 considered the high-exacerbation-risk patients as the E group to replace the GOLD C and D groups; however, the necessity of this replacement should be further explored by the prognosis between the GOLD C and D groups.

When it came to the rate and risk of future exacerbation, our results showed that there was a significant difference between the GOLD C and A or B groups, but no difference between the GOLD C and D groups. Recently, another study indicated that the exacerbation rate within 12 months in the GOLD C group was lower than that in the GOLD D group,^[16]^ where they classified COPD patients mainly based on the CAT, which may have reallocated some patients of the GOLD D group into the GOLD C group. This classification method may result in differences between our study and their research. The difference between the GOLD C and D groups was in the CAT and mMRC scores, and both groups were at high exacerbation risk. The CAT or mMRC scores were largely dependent on each patient’s own feelings and the doctors’ judgment. However, each patient experienced symptoms at different disease severities, and doctors may have a subjective bias regarding the extent of patients’ symptoms, which may result in inaccurate symptom evaluation in high-exacerbation-risk patients. As for the exacerbation history, the answer was simple: include “Yes” or “No” based on occurrence, which is more objective. Previous studies had demonstrated that the exacerbation history was the best predictor of future exacerbation.^[14,15]^

For all-cause mortality, the highest rate was 8.2% for the GOLD D group, followed by GOLD B, C, and A groups at the 3-year follow-up, respectively, which aligns with previous research.^[29,31,33]^ However, the only difference identified was in the risk of 3-year all-cause mortality between the GOLD C and D groups, as determined by univariate COX regression. Still, after adjustment for confounding factors, the risk of all-cause mortality was not significantly different between the GOLD C and D groups. The only difference in all-cause mortality between the GOLD C and D groups may be associated with symptom severity, but inhaled treatment, pulmonary function, body mass index, or other factors may offset the effect of symptoms. Moreover, all-cause mortality included COPD-related and non-COPD-related mortality, and the differing occurrence of these 2 may partly account for the nonsignificant association of all-cause mortality between the GOLD C and D groups in the adjusted COX regression. According to the Kaplan–Meier survival curve, the GOLD C group showed an unstable trend, possibly due to having the fewest patients among all the GOLD groups.

After combining the GOLD C and D groups into the E group, the recommended initial inhaled treatment for the GOLD E group was LABA/LAMA. However, it should be considered whether the GOLD C and D groups had a unique treatment response to inhaled therapy. This study demonstrated that, in the GOLD C group, there was no statistically significant difference in future exacerbation rate or risk across inhaled treatment groups. In contrast, in the GOLD D group, patients treated with LABA/LAMA or LABA/LAMA/ICS exhibited lower rates or risks of any future exacerbation and frequent exacerbation. Previous studies had found that LABA/LAMA was superior to LABA/ICS and LAMA.^[17–20]^ Suissa et al.^[34]^ found that LABA/LAMA and LABA/LAMA/ICS can reduce the risk of exacerbation in COPD patients to the same extent, but among patients without high eosinophilia and a history of frequent exacerbations, LABA/LAMA/ICS was associated with more cases of pneumonia. Thus, our results and previous studies support a change in the GOLD groups, but this study was observational, and this change should be further explored in a randomized controlled trial.

This study has some limitations. First, it was a retrospective study with a relatively small number of patients in the GOLD C group. A statistical power test was performed, with the results indicating a high value (Table S1, Supplemental Digital Content 1), which means there is a lower likelihood of a type II error. Also, the chi-square test, logistic regression, and COX regression with stratified adjustment were used to analyze the prognosis between the GOLD C and D groups. Based on our statistical methods, our results have a certain degree of credibility but should be further validated in a large cohort of patients in the GOLD C group. Second, the number of patients with LABA/LAMA was relatively small due to the late launch of LABA/LAMA in China (in 2000). Third, this study recorded only all-cause mortality; COPD-related or respiratory mortality should be further examined. In the future, large prospective studies across multiple centers should be conducted.

## 5. Conclusion

In COPD, the GOLD C group was the smallest and had similar risks of future exacerbation and all-cause mortality compared to the GOLD D group. Different inhaled treatments showed less impact on the risk of future exacerbation for patients in the GOLD C group, while LABA/LAMA or LABA/LAMA/ICS decreased the risk of future exacerbation for patients in the GOLD D group. The proposal of the GOLD E group to replace the GOLD C and D groups could be considered, and it could simplify the classification of COPD patients.

## Acknowledgments

We would like to thank the hospital staff for their contributions to data collection, especially the research assistants Guolin Zhong from the Second Xiangya Hospital, Central South University.

## Author contributions

**Conceptualization:** Tao Li, Ping Zhang, Ping Chen.

**Data curation:** Tao Li, Ping Zhang, Qing Song, Cong Liu, Ling Lin, Yuqin Zeng.

**Formal analysis:** Tao Li.

**Funding acquisition:** Ping Chen.

**Investigation:** Tao Li.

**Methodology:** Tao Li, Ping Zhang, Qing Song, Cong Liu, Ling Lin, Yuqin Zeng, Ping Chen.

**Project administration:** Tao Li, Ping Zhang, Ping Chen.

**Supervision:** Ping Zhang, Ping Chen.

**Writing – original draft:** Tao Li.

**Writing – review & editing:** Tao Li, Ping Zhang, Qing Song, Cong Liu, Ling Lin, Yuqin Zeng, Ping Chen.


