# Supplementary material for: Is it essential for the combination of GOLD C and D groups in COPD patients: A multicenter retrospective study?
Source: Medicine (Baltimore). 2026 Aug 28;105(35):e50492. doi: 10.1097/MD.0000000000050492 (PMC13529139; doi:10.1097/MD.0000000000050492)
Supplement: Supplementary file 1 [file medi-105-e50492-s001.docx]

Supplement Table 1 The proportion difference test for statistical power of GOLD 2017 C and D groups

| **Statistical Power** | **Risk difference** | **Relative Risk** | **Odds Ratio** | **Significance level** |
| --- | --- | --- | --- | --- |
| **1.000** | -0.355 | 0.080 | 0.051 | 0.050 |

Note: Two-sided large-sample test. Power was estimated by Pearson’s chi-square test with pooled standard deviation and continuity correction.
